# Can Transcutaneous Vagus Nerve Stimulation be Effective After Rats’ Spinal Cord Injury?

**DOI:** 10.7759/cureus.102944

**Published:** 2026-02-04

**Authors:** Tomoko Tanaka, Murat Gokden, Reid D Landes

**Affiliations:** 1 Neurosurgery, University of Arkansas for Medical Sciences, Little Rock, USA; 2 Pathology/Neuropathology, University of Arkansas for Medical Sciences, Little Rock, USA; 3 Biostatistics, University of Arkansas for Medical Sciences, Little Rock, USA

**Keywords:** macrophage, neuroinflammation, recovery, rodent, spinal cord injury, transcutaneous electrical nerve stimulation, vagus nerve stimulation

## Abstract

Introduction: Most spinal cord injuries (SCIs) result from trauma, and despite extensive research, no curative treatment exists. SCIs occur from primary and secondary injuries. The primary injury is caused by direct impact from the initial trauma, mechanical disruption, dislocation, stretch, compression, or ischemia involving the spinal cord. The primary injury leads to a secondary injury, characterized by the sequential progression of cell dysfunction and death, which begins with an influx of inflammatory cells and cytokines and ultimately results in apoptosis, necrosis, and gliosis. Thus, mitigating the neuroinflammatory process has been a key focus in treating further secondary injuries by multiple modalities, which include vagus nerve stimulation (VNS). The present project investigated the effect of transcutaneous VNS (t-VNS) on the inflammatory effects of SCI.

Methods: The histopathology of the SCI contusion model in male Sprague-Dawley rats was compared between groups that received t-VNS and those that did not.

Result: No statistical difference was found between SCI with and without t-VNS in M1, M2a, M2b, and M2c macrophages (all p>0.27). However, post-hoc correlations indicated M1 macrophages declined more rapidly over time in the t-VNS group. (Spearman r= -0.50; 95% CI: -0.78, -0.05).

Conclusions: Although t-VNS did not significantly alter macrophage distribution, trends toward reduced M1 activation suggest a possible anti-inflammatory effect in the desired direction.

## Introduction

Globally, over 15 million people live with spinal cord injury (SCI), most of which results from trauma. In the United States, approximately 2.6 million individuals are affected, with annual hospitalization costs estimated at $1.6-$1.7 billion [1,2]. SCI imposes significant physical, emotional, and financial burdens, motivating ongoing research to improve recovery and quality of life [3,4]. The pathophysiology involves a primary mechanical insult, disruption, compression, or ischemia of the spinal cord, followed by a secondary injury driven by inflammatory cascades leading to cellular dysfunction and death [5,6]. Cytokines such as IL-1, IL-6, and TNF, together with activated macrophages, microglia, and neutrophils, mediate this process [7,8]. Consequently, modulation of macrophage activity has become a therapeutic target [9]. Although splenectomy has been shown to have neuroprotective effects by reducing macrophage infiltration [10,11], it remains clinically impractical. Alternatively, Popovich et al. demonstrated that intravenous clodronate liposomes can deplete hematogenous macrophages and enhance recovery in rodent SCI models [9,12]. 

There are two different types of macrophages, M1 and M2. M1 macrophages are essentially harmful to the central nervous system, while M2 macrophages act in an anti-inflammatory capacity [13]. Suppressing M1 and endorsing M2 to mitigate central nervous system attack and encourage neuroprotection is a concept called polarization of macrophages. Research into the polarization of macrophages and microglia has advanced targeted neuroprotection through the delivery of molecules to alter the phenotype of macrophages/microglia, the transplant of mesenchymal stem cells, or the direct SCI-site delivery of microRNAs that regulate macrophage/microglia polarization [14]. 

Various technologies have attempted neuromodulation to promote recovery of the injured SCI via the electrically stimulated brainstem, corticospinal, and reticulospinal tracts [3]. Tracy, Bonaz, Bloom, and Bonaz [8,15-17] have described the impact of the vagus nerve on the inflammatory reflex. Vagus nerve stimulation (VNS) has been investigated for various pathologies, including neurological and psychological diseases, immune diseases, cardiovascular disease, chronic pain, and other autonomic dysfunctions [18]. 

The vagus nerve is the longest nerve in the body and is the major output of the parasympathetic nervous system. Approximately 80% of vagal nerve fibers are afferent, transmitting visceral sensory information to the nucleus tractus solitarius, which modulates hypothalamic-pituitary-adrenal (HPA) activity, and 20% efferent (cholinergic anti-inflammatory pathway). The efferent vagus nerve originates in the brainstem's dorsal motor nucleus of the vagus, and the nucleus ambiguus innervates the heart, lungs, and many other visceral organs that regulate metabolism and homeostasis [8,15]. The vagus nerve modulates splenic immune responses indirectly via the celiac-superior mesenteric ganglion and splenic nerve.

The US Food and Drug Administration (FDA) has approved surgically implanted vagus nerve stimulation (VNS) for treating epilepsy and transcutaneous VNS (t-VNS) for migraines [19]. Most research on VNS in the context of spinal cord injury (SCI) has involved implanted devices; however, implanting VNS carries risks, including vocal cord paralysis, infection, and cardiac arrhythmia [20]. Given the potential for future clinical trials and the desire for a less invasive method with similar effectiveness, it is important to explore non-surgical methods. t-VNS, a non-invasive technique to deliver electrical pulses transcutaneously to the vagus nerve [21,22]. This approach is rooted in both Eastern and Western medical practices, particularly auricular acupuncture [23,24], which has been recognized for its health benefits. We hypothesize that t-VNS could reduce inflammatory reflexes in SCI by shifting macrophage distributions, increasing M2-type macrophages (anti-inflammatory), and decreasing M1-type macrophages (pro-inflammatory). 

## Materials and methods

Animals 

Male Sprague-Dawley rats, 8 to 9 weeks old and weighing 180 to 300 g, were purchased from Charles River Laboratories (Memphis, TN). These ages in rats correspond to the ages of adolescent and young adult humans. This study was approved by the local Institutional Animal Care and Use Committee under protocol #4074. 

A total of 39 rats were allocated to two treatment groups: SCI without t-VNS (SCI-only) and SCI with t-VNS (SCI + t-VNS). SCI involved surgically exposing the spinal cord (laminectomy), followed by mechanical contusion (see below). We originally planned sham groups of laminectomies with and without t-VNS. The first several sham rats showed no pathological findings or macrophages. Thus, we terminated the sham groups for further investigation to decrease the total number of experimental rats (Table 1).

**Table 1:** Designed and experiment groups of projects SCI: Spinal cord injury, t VNS: transcutaneous vagus nerve stimulation

Group #	Initial Designed Procedure	Actual Experiment
1	Sham SCI without t VNS	N/A
2	Sham SCI with t VNS	N/A
3	SCI without t VNS	SCI without t VNS
4	SCI with t VNS	SCI with t VNS

More details of the experiment designs are in the Appendix.

Spinal cord injury

Rats were anesthetized (0.5-1.0 L/min flow) with 2-5% isoflurane. After shaving the hair off the surgical area on the rat’s lower back with electric clippers, an iodine preparation was applied. SCI was accomplished through a laminectomy followed by spinal contusion. First, a laminectomy was performed at T12/L1 to expose the spinal cord, after which rats were clamped into the spinal fixation device. Then, a custom spinal cord impactor device (Model III; Keck Center for Collaborative Neuroscience, Piscataway, NJ) with a 3.0 mm diameter tip was centered perpendicularly over the spinal cord and used to deliver a spinal cord contusion by an impactor height of 25 mm at a velocity of 0.7 m/sec.

The extent of spinal cord injury was confirmed with direct visualization of swelling of the spinal cord and discoloration, in addition to the impactor outcome on computer digital analysis.

Transcutaneous vagus nerve stimulation 

**Figure 1:**
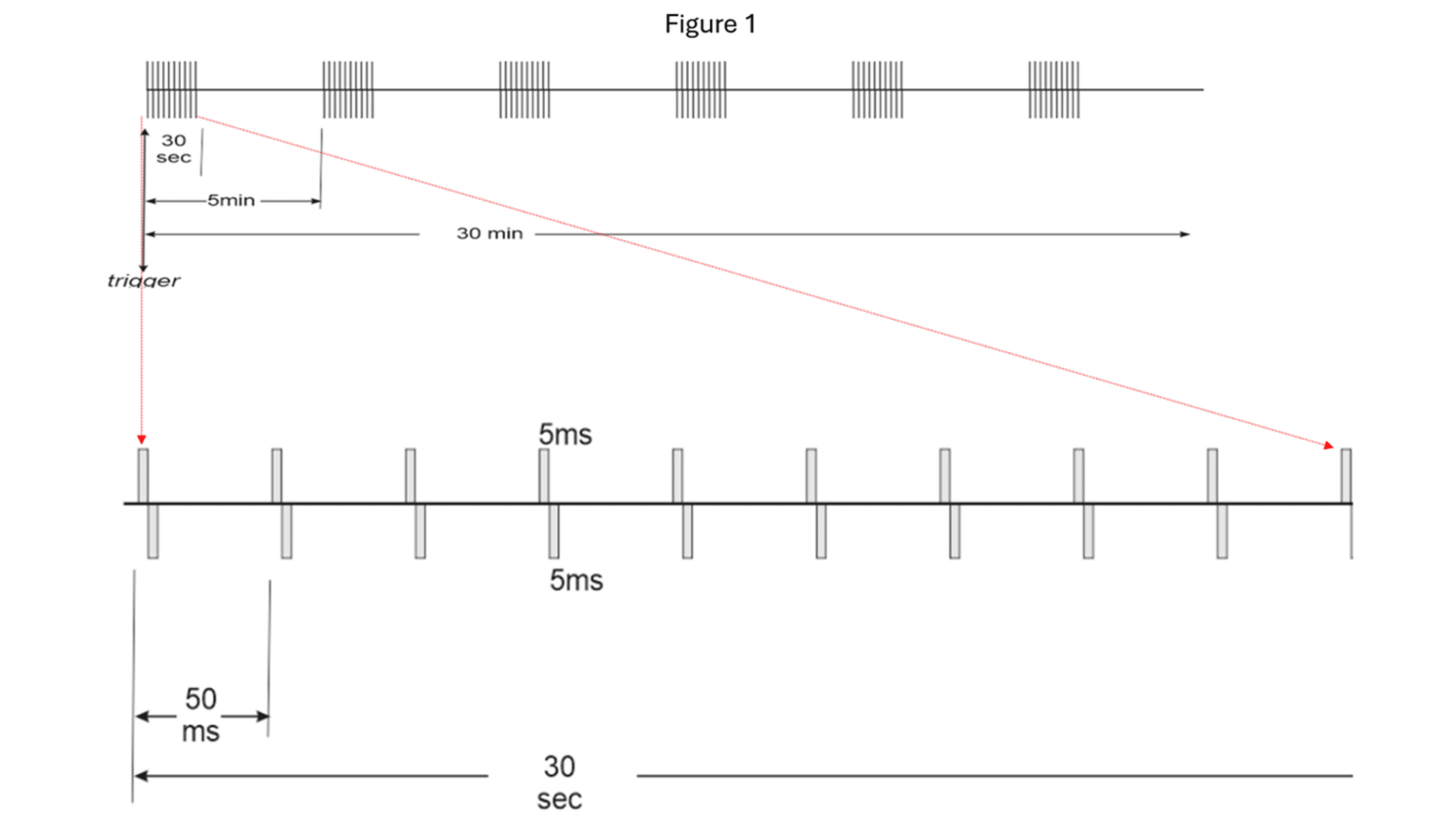
Schematic view of vagus nerve stimulation protocol The upper schematic shows a 30-second train of stimulation consisting of 0.5-ms biphasic square pulses (0.5 mA) delivered at 20 Hz, initiated for 30 minutes. The lower schematic shows the details of a 30-second train of stimulation.

After closing the incision, the SCI + t-VNS group was subjected to t-VNS by an AM model 4100 portable, isolated high-powered stimulator (A-M system, Sequim, WA). Two acupuncture needles were inserted into the rats, one in the cymba concha and cavum concha region of the ear and the second in the retroauricular region behind the ear. Alligator clips were hooked to each of the acupuncture needles to allow t-VNS stimulation to be programmatically delivered. The treatment consisted of 30-second stimulations consisting of 0.5 ms, 0.5 mA bi-phasic square pulses delivered at 20 Hz, followed by a 4.5-minute rest period, repeated over the course of 30 minutes. Given that the parameters of VNS, including duration, frequency, and pulse width, vary across studies, we adopted a stimulation protocol based on previously established models in which t-VNS was applied to rats with traumatic brain injury [25] and to rats with stroke [26]. Because the acupuncture needles needed to stay in position, and the discomfort of electric stimulation, the t-VNS group remained under anesthesia for the duration of stimulation. (Schematic view of protocol: Figure 1).

Post-operative care 

After the experiment, rats were allowed to recover completely from anesthesia and then returned to their cage. All rats confirmed bilateral hind limb weakness after surgery. All postoperative rats received an initial postoperative dose of buprenorphine (SQ) 12 hours following anesthetic recovery. The following day, each rat was observed twice daily to ensure their health until euthanasia. The rats were housed in cages lined with approximately twice the amount of standard bedding material to prevent the development of dermal ulceration. The rats that had distension due to a neurogenic bladder were manually expressed by applying pressure to the abdomen twice a day until the scheduled euthanasia day. When the rat's wellness was concerned, onsite veterinary staff members were contacted to assess the rat's condition. A veterinary staff member was consulted to assess a surgical site hematoma on one rat, but euthanasia was not required. 

Histology and macrophage rating 

**Figure 2:**
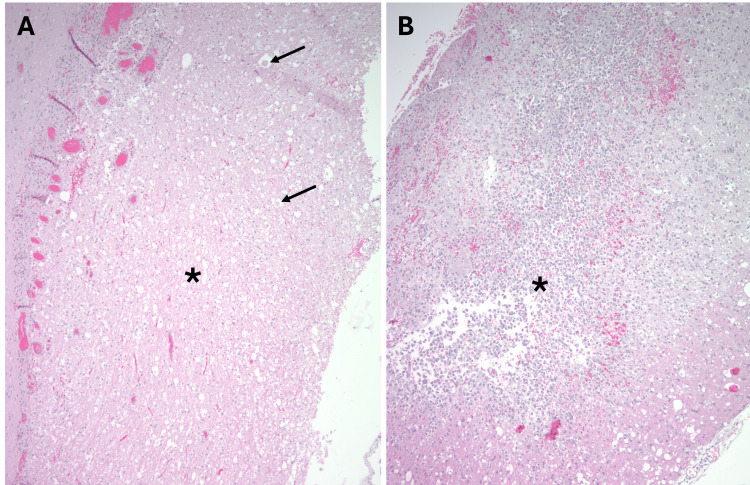
Examples of SCI on hematoxylin and eosin (H&E) Acute infarct (*) with edema and axonal spheroids (arrows; A). Subacute infarct with a dense infiltrate of histiocytes (*; B). (Hematoxylin and eosin; original magnification: A and B 100x)

**Figure 3:**
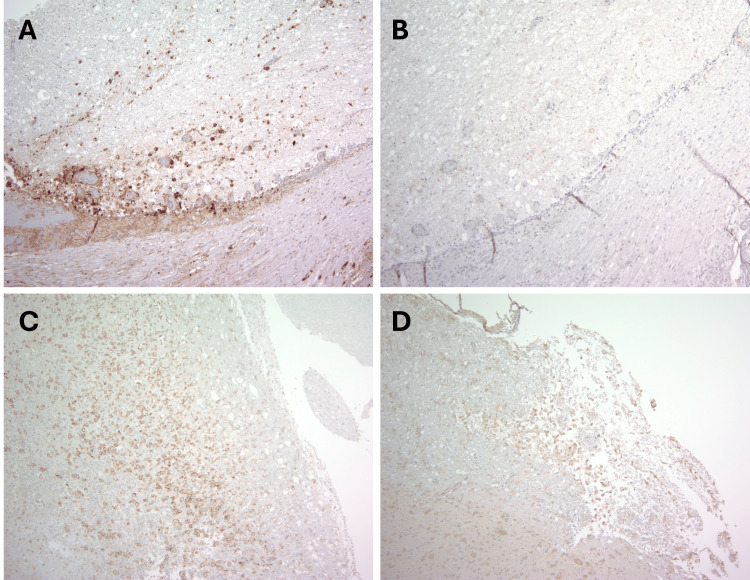
Examples of SCI on immunochemical stains An example of an acute infarct with arginase 1-positive histiocytes (A) and no CD16-positive histiocytes (B). An example of a subacute infarct with abundant CD163-positive histiocytes (C) and another with CD64-positive histiocytes (D). (Immunohistochemistry; original magnifications: A–D 100x). SCI: Spinal cord injury

Seventy-two to one hundred forty-four hours after the procedure, animals were euthanized (Hours: Number of rats = 72: 8, 96: 6, 120: 18, 144: 4). Then, a level laminectomy was performed to remove the spinal cord, which was placed in 4% paraformaldehyde for fixation and submitted for standard tissue processing and paraffin embedding. Four micrometer-thick paraffin sections were prepared and stained with hematoxylin and eosin. Immunohistochemical stains for CD16, arginase 1, CD64, and CD163 (Abcam Inc., Waltham, MA) for M1, M2a, M2b, and M2c, respectively. Slides were evaluated by a neuropathologist (MG) who was blinded to the treatment information and used a semi-quantitative method for rating the infiltrates. The amount of each subgroup of macrophages on a slide was scored 0 - 4 (ordinal values), indicating the least to greatest amounts. Score 0 was used if no or only a rare positive cell is present. Scores 1-4 ranked the four macrophage subgroups from the least frequent to the most frequent in a given spinal cord. Examples of cases stained with hematoxylin and eosin (H&E) and immunohistochemistry are shown in Figures 2, 3, respectively.

Statistical analysis 

We used Mann-Whitney U-tests to compare ratings between the two groups. Post-hoc, we also calculated Spearman's rank correlation to evaluate the relationship of ratings to time after SCI. Individual rats were the experimental units. More details of the planned and enacted statistical and power analyses are in the Appendix. We refer to results with p < 0.05 as statistically significant.

## Results

**Figure 4:**
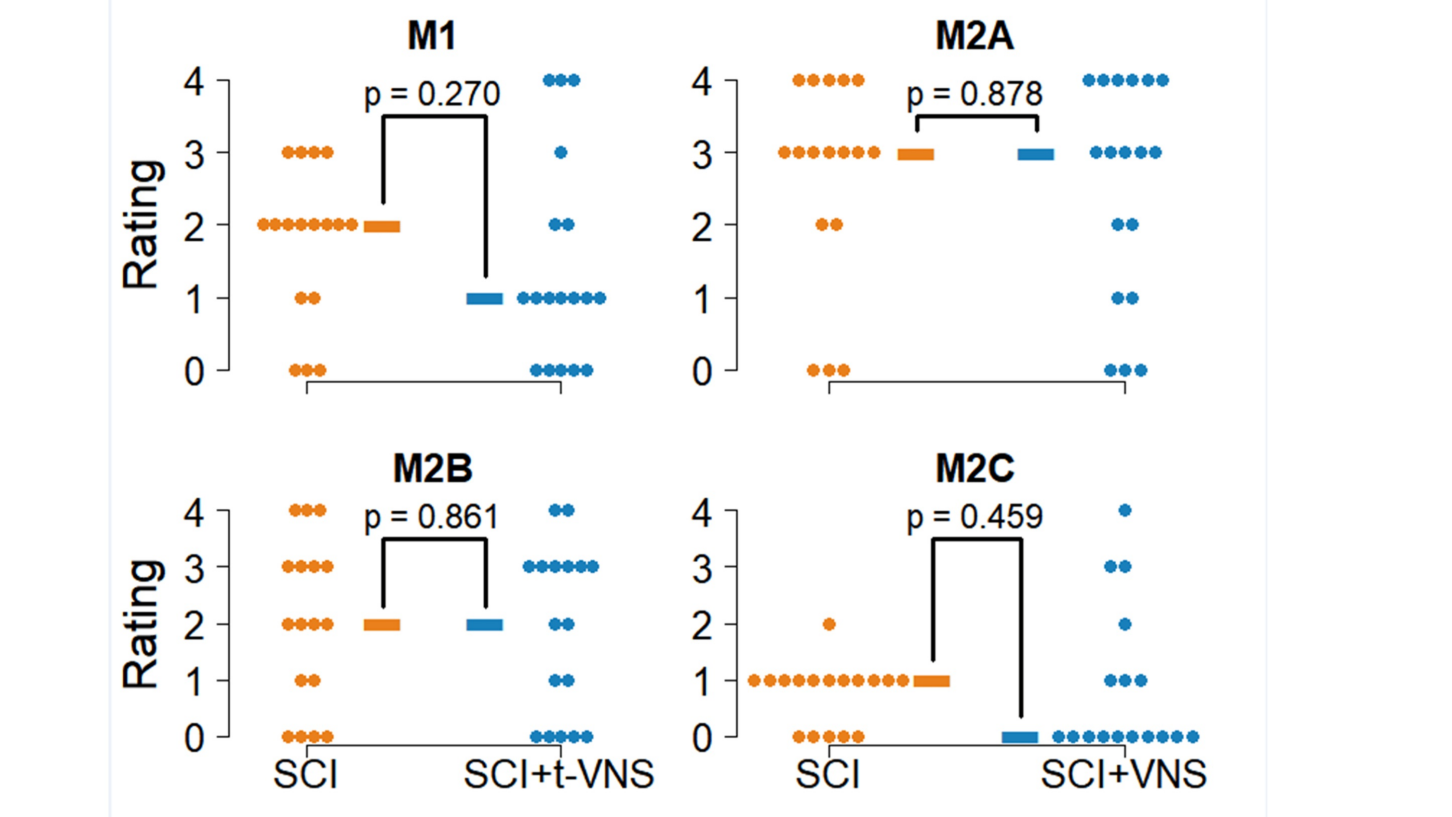
Results of macrophage distribution after SCI Macrophage rating scores for (A) M1, (B) M2A, (C) M2B, and (D) M2C macrophages. Individual scores are plotted with filled circles and medians with a dash. Medians were compared with Wilcoxon rank sum test. Orange is for SCI alone, and blue is for SCI + t-VNS. SCI: Spinal cord injury

We performed a laminectomy followed by SCI on 39 rats. Four rats died during the process. Therefore, we collected data from 17 rats in the SCI-only group and 18 in the SCI + t-VNS group. Our pre-specified primary comparison was of the macrophage distributions between sham and sham with t-VNS rats, without regard to time since SCI. None of the macrophage distributions statistically differed between sham and sham-with-t-VNS rats. The M1 macrophages were estimated to be lower in t-VNS animals as hypothesized. However, M2 macrophages in SCI with t-VNS rats were no higher than in SCI without t-VNS rats; thus, the differences were not in the hypothesized direction (Figure 4).

**Figure 5:**
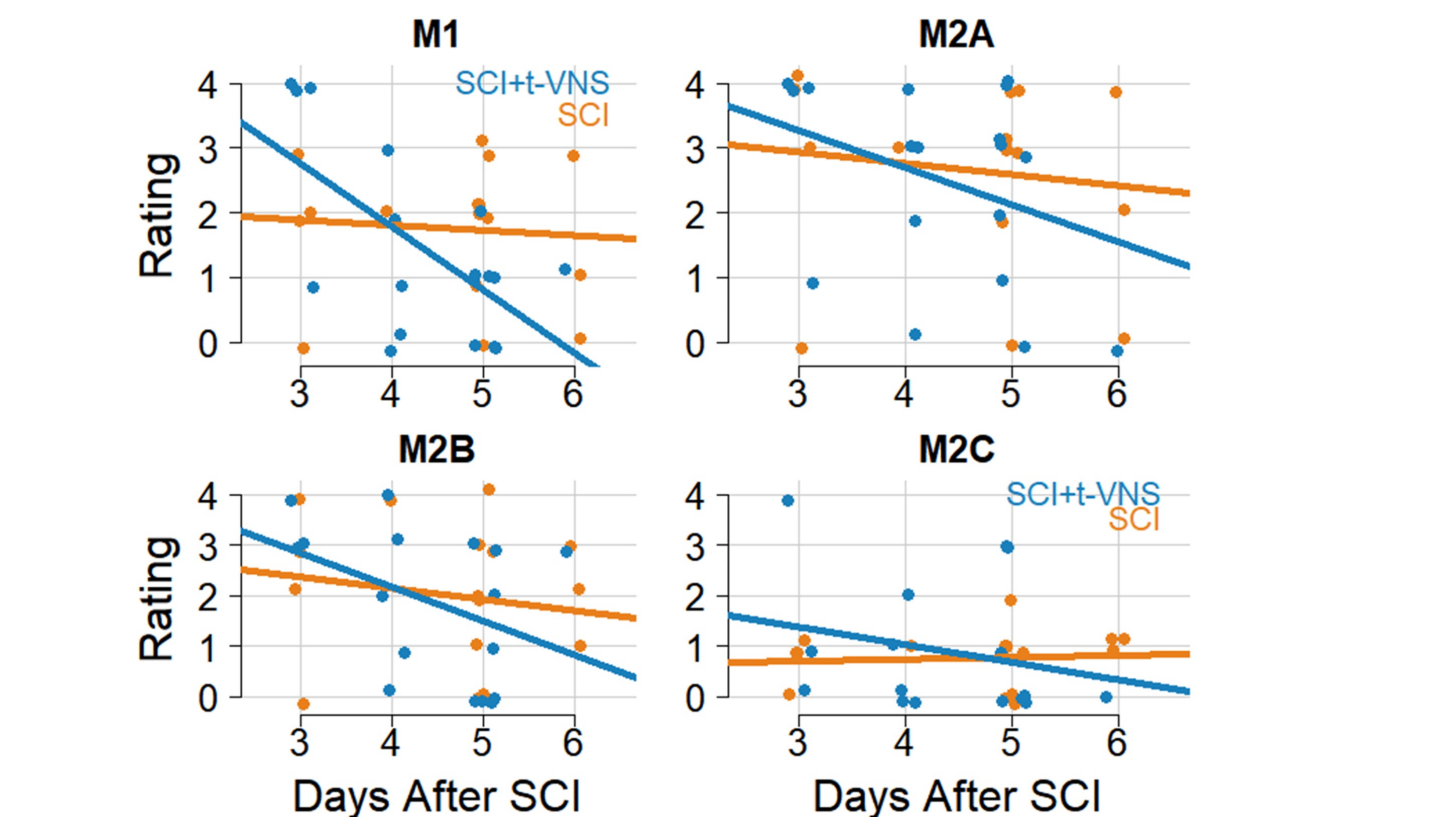
Results of macrophage distribution over time since SCI Macrophage rating scores for (A) M1, (B) M2A, (C) M2B, and (D) M2C macrophages are plotted by days after SCI with best-fit lines; see Table 2 for rank correlations. Orange is for SCI alone and blue for SCI + t-VNS.

We correlated the macrophage scores with days since SCI within both groups (Table 2); this was a post-hoc analysis. For SCI + t-VNS rats, M1 macrophages decreased as days since SCI increased (Spearman r = -0.50; 95% CI: -0.78, -0.05; Figure 5A). We did not find any other significant correlation. Additionally, when comparing correlations between the SCI-only and SCI + t-VNS groups, we found no differences within the macrophage types (Ps > 0.270). 

**Table 2:** Spearman correlations (r) of cell type rating scores and days since SCI Spearman r = -0.50; 95% CI: -0.78, -0.05. a: For SCI + t VNS, one rat’s specimen could not be scored for M2B and M2C macrophages.

	SCI + t VNS (n = 18)		SCI alone (n = 17)	
Cell type	r	p-value	r	p-value
M1	-0.104	0.692	-0.501	0.034
M2A	-0.199	0.443	-0.350	0.155
M2B^a^	-0.176	0.499	-0.408	0.104
M2C^a^	0.083	0.752	-0.231	0.371

## Discussion

In this study, we compared M1/M2 macrophage distributions following SCI in rats treated with t-VNS versus SCI alone. Among the 35 animals (17 SCI-only, 18 SCI + t-VNS), median macrophage subtype scores did not differ significantly between groups. However, post-hoc analysis demonstrated a reduction in M1 macrophages in the SCI + t-VNS group. Although these differences did not reach overall statistical significance, we observed a notable trend toward earlier and reduced expression of neurotoxic M1 macrophages, accompanied by earlier appearance of M2 macrophage subtypes following t-VNS. These findings suggest that t-VNS may influence post-injury inflammatory dynamics, even with a single stimulation session.

Our observations are consistent with prior work by Chen et al. [27], who demonstrated that implanted cervical vagus nerve stimulation promoted macrophage polarization and improved functional recovery after SCI. While this supports the general hypothesis that vagus nerve stimulation can modulate inflammatory reflexes, direct comparison between studies is limited by methodological differences. Specifically, implanted VNS with repeated daily stimulation over 14 days, whereas our study employed a single 30-minute session of t-VNS.

In addition, t-VNS was applied immediately after SCI under general anesthesia, as repeated stimulation in awake animals was not feasible. The effects of general anesthesia on neuro-immune interactions remain unknown [28] and may have confounded our results by altering inflammatory signaling pathways. Furthermore, our study focused exclusively on macrophage polarization and did not include functional behavioral assessments, such as motor recovery evaluated using the Basso, Beattie, and Bresnahan (BBB) locomotor rating scale [29]. The absence of functional outcome measures limits our ability to directly link observed immunological changes with neurological recovery.

Another limitation is that macrophage phenotypes were characterized using a limited set of histological markers. Macrophage polarization represents a dynamic spectrum regulated by cytokines and chemokines [30], and we did not perform multi-marker immunostaining or cytokine profiling. Future studies incorporating pro-inflammatory cytokines (e.g., TNF-α, IL-1β, IL-6), anti-inflammatory cytokines (e.g., IL-10), and multiplex staining approaches would allow more comprehensive validation of macrophage functionality and polarization states.

Despite these limitations, our findings provide preliminary support for the feasibility and potential immunomodulatory effects of t-VNS following SCI. The non-invasive, portable, and clinically accessible t-VNS makes it an attractive therapeutic candidate for SCI, particularly if optimized through repeated stimulation protocols and combined with functional outcome assessments. Larger studies incorporating longitudinal behavioral analysis and comprehensive immunophenotyping will be essential to determine the therapeutic efficacy and translational relevance of t-VNS for SCI recovery.

## Conclusions

Although statistical significance was not achieved, the observed rapid reduction in M1 activity following t-VNS provides encouraging preliminary evidence supporting our hypothesis. These findings underscore the therapeutic promise of portable, non-invasive vagus nerve stimulation and justify further investigation in larger, adequately powered studies to determine its potential to enhance recovery following spinal cord injury. Our findings are preliminary and should be interpreted with caution.

## Appendices

Transparency, rigor, and reproducibility

We had intended to experiment with males and females, but there was a mistake in the order, and we only received males. The sample size was calculated a priori; a complete description is found in Table 3. We did not perform a proper randomization of treatment order within the surgery day as initially intended, but we did alternate between treatment with and without t-VNS on most days. Regarding the discontinuation of the two sham-SCI groups, there were no macrophages in the tissue for the neuropathologist to score from rats subjected to sham SCI. Because these animals could not provide data for analysis, we stopped investigating those groups. The checklist for the ARRIVE 2.0 Essential 10 is included in the supplementary material.

**Table 3:** Source data

ID	GROUP	SEX	AGE	WT	SCI_DATE	END_DATE	DAYS_AFTER	M1	M2A	M2B	M2C	ORIG_ORDER	NOTE1
14	1	Male	8	299	1/14/2022	1/18/2022	4	0	0	0	0	1	
15	2	Male	8	287	1/14/2022	1/18/2022	4	0	0	0	0	2	
16	4	Male	8	280	1/14/2022	1/18/2022	4	1	4	4	2	3	
17	3	Male	8	292	1/14/2022	1/18/2022	4	2	3	4	1	4	
18	2	Male	9	280	1/21/2022	1/25/2022	4	0	0	0	0	5	
19	4	Male	9	272	1/21/2022	1/25/2022	4	3	2	2	1	6	
20	4	Male	9	290	1/21/2022	1/25/2022	4	0	3	3	0	7	
21	4	Male	9	202	1/21/2022	1/25/2022	4	2	3	1	0	8	
22	4	Male	9	262	1/21/2022	1/25/2022	4	0	0	0	0	9	
23	4	Male	8	180	2/2/2022	2/7/2022	5	1	3	1	0	10	
24	3	Male	8	200	2/2/2022	2/7/2022	5	1	3	2	0	11	
25	4	Male	8	195	2/2/2022	2/7/2022	5	1	3	0	0	12	
26	3	Male	8	191	2/2/2022	2/7/2022	5	0	0	0	0	13	
27	4	Male	8	186	2/2/2022	2/7/2022	5	0	2	3	0	14	
28	3	Male	8	188	2/2/2022	2/7/2022	5	2	2	4	0	15	
30	3	Male	8	190	2/2/2022	2/7/2022	5	2	3	2	0	16	
31	4	Male	8	188	2/2/2022	2/7/2022	5	1	3	2	0	17	
32	3	Male	8	186	2/2/2022	2/7/2022	5	3	3	1	1	18	
33	2	Male	8	213	2/16/2022	2/22/2022	6	0	0	0	0	19	
34	1	Male	8	218	2/16/2022	2/22/2022	6	0	0	0	0	20	
36	3	Male	8	203	2/16/2022	2/22/2022	6	1	0	1	1	21	
37	2	Male	8	195	2/16/2022	2/22/2022	6	0	0	0	0	22	
38	3	Male	8	215	2/16/2022	2/22/2022	6	0	2	3	1	23	
39	4	Male	8	217	2/16/2022	2/22/2022	6	1	0	3	0	24	
40	3	Male	8	200	2/16/2022	2/22/2022	6	3	4	2	1	25	
41	2	Male	8	215	2/16/2022	2/22/2022	6	1	2	4	2	26	Surgeon: Trauma from sham surgery
42.1	1	Male	8	224	2/16/2022	2/22/2022	6	0	3	1	1	27	Statistician: Somehow pathology received #42 in different day; updating ID number
42.2	2	Male	8	222	4/29/2022	5/4/2022	5	0	0	0	0	28	Statistician: Somehow pathology received #42 in different day; updating ID number
43	1	Male	8	210	4/29/2022	5/4/2022	5	0	1	1	1	29	
44	4	Male	8	200	4/29/2022	5/4/2022	5	0	0	0	0	30	
45	3	Male	8	196	4/29/2022	5/4/2022	5	2	3	3	1	31	
46	4	Male	8	213	4/29/2022	5/4/2022	5	2	4	3	3	32	
47	3	Male	8	219	4/29/2022	5/4/2022	5	2	3	3	2	33	
48	4	Male	8	220	4/29/2022	5/4/2022	5	1	1	0	1	34	
49	3	Male	8	201	4/29/2022	5/4/2022	5	2	4	0	1	35	
50	4	Male	8	210	4/29/2022	5/4/2022	5	0	4	0	3	36	
51	3	Male	8	200	4/29/2022	5/4/2022	5	3	4	0	1	37	
52	2	Male	9	211	5/24/2022	5/27/2022	3	4	3	4	0	38	Surgeon: Trauma from sham surgery
53	1	Male	9	236	5/24/2022	5/27/2022	3	0	0	0	0	39	
54	4	Male	9	210	5/24/2022	5/27/2022	3	4	4	4	4	40	
55	3	Male	9	222	5/24/2022	5/27/2022	3	2	4	3	1	41	
56	4	Male	9	224	5/24/2022	5/27/2022	3	4	4	3	0	42	
57	3	Male	9	213	5/24/2022	5/27/2022	3	0	0	0	0	43	
58	4	Male	9	201	5/24/2022	5/27/2022	3	1	1			44	Neuropathologist: Tissue amount was very small and no specimen could be seen for CD64 & CD-163.
59	3	Male	9	228	5/24/2022	5/27/2022	3	2	3	2	1	45	
60	4	Male	9	213	5/24/2022	5/27/2022	3	4	4	3	1	46	
61	3	Male	9	209	5/24/2022	5/27/2022	3	3	4	4	1	47	

Data availability

The source data and statistical software code used to analyze them are included in the Statistical and Power Analyses section of the Appendix.

Experimental Design

Planned: the treatment groups were to be all four combinations of SCI (sham & active) and VNS (none & active). On any given surgery day, we anticipated completing the experimental procedures on up to 4 rats. Hence, we planned to randomize the four treatments among the four rats for the day. Surgery day was to be treated as a random blocking factor.

Enacted: we discontinued the sham-SCI groups after completing 5 rats in one group and 7 in another; see the explanation in Transparency, Rigor, and Reproducibility. Thus, we had only 2 groups, SCI + no-t-VNS and SCI + t-VNS, going forward. On most days, we alternated the t-VNS application on every other SCI rat. On one day, we did not have any SCI + no-t-VNS rats; just SCI + t-VNS. The number of SCI rats was not limited to four on any given surgical day: the number of SCI rats undergoing procedures ranged from 2 to 9.

Statistical and Power Analyses

Planned: we assumed the outcomes would be normally distributed; hence, we planned to use an ANOVA that accounted for treatment group (a fixed effect) and surgery day (a random effect). The primary comparison of interest was planned to be between SCI alone and SCI + t-VNS; we based our sample size calculations on this comparison. These comparisons were to be conducted with t-tests within the ANOVA context.

Sample size calculations: we based our calculations on flow cytometry data from Figure 4B in Hellenbrand et al. [7]. In their experiment, their control is analogous to our SCI alone group. They also had four active treatments for SCI. The differences in means between the control and each of the active treatments ranged from 3.4 to 10.9 percentage points. The standard deviations (SDs) for the active treatment groups ranged from 5.0 to 8.5 percentage points. We assumed an SD of 7 percentage points for all of our groups.

We hoped to detect with at least 0.80 power whether active t-VNS treatment will reduce the percentage of M1 macrophages in animals with SCI by at least 1 SD (i.e., 7 percentage points). Using a one-sided 0.05 level test, we would need 13 rats/group (26 rats in total).

Enacted: the primary outcome variable changed from flow cytometry measures to the semi-quantitative rating by the neuropathologist. Though we collected the surgery day data, when the neuropathologist rated the samples, he did so all at one sitting, and, importantly, rated the samples without regard to procedure day. Because of this, we dropped surgery day as a potential blocking variable. Further, because the ratings were ordinal, we use Wilcoxon-Mann-Whitney test to compare the two groups. Additionally, the rats were not all sacrificed at the same time point after surgery; rather, the sacrifice day, post-surgery, was based on sequala of unforeseen clinical obligations that came up at planned sacrificed times. As a physician-scientist, clinical duties were weighted more than research activities. Having the time between surgery and sacrifice added useful information. We decided to correlate the ratings (of each cell type) with the number of days since surgery; we used Spearman’s correlations because of the ordinal ratings. However, for Figure 2, we illustrated the relationships of the ratings with simple linear regressions.

Data and Statistical Software

The source data are part of the supplemental files, entitled “SCI_and_tVNS_source_data.xlsx”. The statistical software code is part of the supplemental files, entitled “SCI_and_t-VNS_SAS_code”.
